# Genome Sequencing of Undiagnosed European Patients Suspected of Hereditary Cancer: Diagnostic Yield and Identification of Candidate Causative Variants

**DOI:** 10.1200/PO-25-00354

**Published:** 2026-04-16

**Authors:** Nelson Martins, Mariona Terradas, José Garcia-Pelaez, Anna K. Sommer, German Demidov, Leslie Matalonga, Mireia Ramos-Muntada, Iris B.A.W. te Paske, Isabel Spier, Arjen Mensenkamp, Janneke Schuurs-Hoeijmakers, Irene Gullo, Celina São José, Ana Maria Pedro, Raquel Gouveia Silva, Ana Berta Sousa, Pedro Amoroso Canão, Susana Fernandes, Luzia Garrido, Juliette Dupont, Sofia Maia, Gabriela Sousa, Arvids Irmejs, Valeria Barili, Ana Blatnik, Paula Rofes, Joan Brunet, Gabriel Capellá, Steven Laurie, Conxi Lázaro, Nicoline Hoogerbrugge, Richarda M. de Voer, Stefan Aretz, Carla Oliveira, Laura Valle

**Affiliations:** ^1^ i3S - Instituto de Investigação e Inovação em Saúde da Universidade do Porto, Porto, Portugal; ^2^ Ipatimup, Institute of Molecular Pathology and Immunology of the University of Porto, Porto, Portugal; ^3^ Hereditary Cancer Program, Catalan Institute of Oncology, IDIBELL, Hospitalet de Llobregat, Barcelona, Spain; ^4^ Program in Molecular Mechanisms and Experimental Therapy in Oncology (Oncobell), IDIBELL, Hospitalet de Llobregat, Barcelona, Spain; ^5^ Centro de Investigación Biomédica en Red de Cáncer (CIBERONC), Madrid, Spain; ^6^ Cell Biology, Physiology and Immunology Department, Faculty of Medicine, Universitat Autònoma de Barcelona, Cerdanyola del Vallès, Spain; ^7^ Faculty of Medicine of the University of Porto (FMUP), Porto, Portugal; ^8^ Institute of Human Genetics, Medical Faculty, University of Bonn, Bonn, Germany; ^9^ Institute of Medical Genetics and Applied Genomics, University of Tübingen, Tübingen, Germany; ^10^ Centro Nacional de Análisis Genómico (CNAG), Barcelona, Spain; ^11^ Universitat de Barcelona (UB), Barcelona, Spain; ^12^ Department of Human Genetics, Radboud University Medical Center, Radboud Research Institute for Medical Innovation, Nijmegen, the Netherlands; ^13^ National Center for Hereditary Tumour Syndromes, University Hospital Bonn, Bonn, Germany; ^14^ RISE-HEALTH, Department of Pathology, Medical Faculty of University of Porto, Porto, Portugal; ^15^ Unidade Local de Saúde de São João (ULSSJ), Porto, Portugal; ^16^ Genetics Service, Unidade Local de Saúde Santa Maria (ULSSM), Centro Académico de Medicina de Lisboa, Lisbon, Portugal; ^17^ Portuguese Oncology Institute of Coimbra, Coimbra, Portugal; ^18^ Institute of Oncology, Riga Stradins University, Riga, Latvia; ^19^ Breast Unit, Pauls Stradins Clinical University Hospital, Riga, Latvia; ^20^ Medical Genetics, Department of Medicine and Surgery, University of Parma, Parma, Italy; ^21^ Department of Clinical Cancer Genetics, Institute of Oncology Ljubljana, Ljubljana, Slovenia; ^22^ Catatan Institute of Oncology, IDIBGi, Girona, Spain

## Abstract

**PURPOSE:**

Hereditary cancers represent 5%-10% of all cancers, typically characterized by familial aggregation, early onset, and/or multiple primary tumors. Isolated cases with extreme early-onset or multiple unrelated cancers are rare and frequently underdiagnosed. This study aimed to improve genetic diagnostic yield in unresolved patients with strong clinical suspicion of hereditary cancer. Inclusion criteria were (1) ≥4 primary tumors in different organs (or ≥3 if two are rare), (2) adult-type cancers at ≤25 years, or (3) profuse gastrointestinal adenomatous polyposis before age 50 years or profuse polyposis of unknown type before age 30 years.

**METHODS:**

Germline DNA from 98 patients selected through ERN-GENTURIS underwent short-read whole-genome sequencing (WGS). Ninety had received negative results from standard genetic testing, whereas eight had not been tested. Variant analysis was performed using the RD-Connect GPAP and Solve-RD frameworks.

**RESULTS:**

Pathogenic or likely pathogenic variants in phenotype-related high-penetrance genes were found in 6% of patients, including *APC* and *TP53* mosaicisms and germline variants in *TP53*, *BAP1*, *BARD1*, and *MBD4* (homozygous). Three patients carried pathogenic variants in hereditary cancer genes unrelated to their phenotype, and a heterozygous *ERCC3* pathogenic variant was detected in a young patient with breast cancer. Suggestive variants of uncertain significance were identified, including a chromosome 3 inversion affecting *VHL* and *MLH1* in a patient with renal and gastric cancers. Potentially regulatory noncoding variants in phenotype-associated genes were observed in 15% of patients (22 variants).

**CONCLUSION:**

Comprehensive resequencing of patients suspected of hereditary cancer increased the diagnostic yield by 6%. Detected pathogenic variants involved phenotype-related genes not previously analyzed or missed because of mosaicism. WGS further enables identification of novel gene associations and structural, deep intronic, or regulatory variants.

## INTRODUCTION

Hereditary tumor predisposition syndromes contribute to 5%-10% of all diagnosed cancers.^1,2^ These syndromes, autosomal dominant or recessive, are caused by germline pathogenic variants (gPVs) in tumor suppressor genes, genes involved in DNA repair, and oncogenes. To date, more than 100 high-/moderate-penetrance genes have been associated with cancer predisposition.^3^

**CONTEXT**

**Key Objective**
To evaluate whether short-read whole-genome sequencing (WGS) improves genetic diagnosis in patients with strong hereditary cancer suspicion who remain undiagnosed after standard testing. This study explores the potential of WGS in detecting coding, noncoding, and structural variants missed by prior conventional methods.
**Knowledge Generated**
WGS identified (likely) pathogenic variants in phenotype-related high-penetrance genes in 6 of 98 patients (approximately 6%), including *APC* and *TP53* mosaicisms, and variants in genes not previously tested. It also uncovered a likely pathogenic *MSH6* variant in a patient with thyroid cancer, and a chromosome 3 inversion of uncertain significance affecting *VHL* and *MLH1* in a patient with renal and gastric cancers. Potential regulatory variants were found in phenotype-related genes.
**Relevance**
More comprehensive resequencing of unresolved hereditary cancer cases increases diagnostic yield by detecting variants in genes not included in the original testing, missed mosaicisms, structural alterations, and, potentially, deep intronic splicing and/or regulatory variants, enabling more accurate diagnoses and personalized management.

Hereditary cancer typically presents with familial clustering, early-onset disease, and/or multiple primary tumors, prompting genetic evaluation.^4^ However, individuals without a family history but showing extreme phenotypes—such as multiple tumors in distinct organs, rare tumor types, or unusually early-onset cancers—often go unrecognized, despite a high likelihood of an underlying genetic predisposition.^5-8^ Even with standard multigene panel testing, many clinically suggestive cases remain without a molecular diagnosis.

The Solve-RD project, funded by the European Commission, aims to provide genetic diagnoses for unsolved patients with rare disease by bringing together six European Reference Networks (ERN), including ERN GENTURIS, which focuses on hereditary cancer.^9-11^ In this context, we performed germline short-read whole-genome sequencing (WGS) on 98 unsolved unrelated patients with suggestive hereditary cancer phenotypes. Our goal was to identify undetected coding and noncoding variants that could explain their clinical phenotypes.

## METHODS

### Patients: Selection Criteria and Cohort Description

Patients were selected based on clinical criteria defined by the ERN GENTURIS Solve-RD Data Interpretation Task Force to identify individuals with strong clinical suspicion of hereditary cancer: (I) Multiple primary cancers in ≥4 organs or ≥3 if two were rare (excluding skin, cervical, and smoking-related lung cancers because of their predominantly nongenetic etiologies), or (II) adult-type cancer diagnosed at age ≤25 years, or (III) ≥50 GI adenomatous polyps diagnosed at age ≤50 years or ≥50 polyps of any histology diagnosed at age ≤30 years (Table 1).

**TABLE 1.** Clinical Criteria and Characteristics of the Patients Included in the Study

Selection Criteria	No. of Patients	No. of Female Patients, %	Median Age at First Cancer Diagnosis, Years (range)	No. of Phenotypes	No. of Individual Phenotypes	Average No. of Phenotypes per Patient
I. Multiple primary cancers affecting at least four different organs or three different organs if two of the cancers are rare^a^	9	7 (77.8%)	36 (11-67)	46	28	4.8
II. Diagnosis of an adult-type cancer before age 25 years	70	59 (84.2%)	22.5 (0.7-25)	95	24	1.2
III. Diagnosis of ≥50 gastrointestinal adenomatous polyps at age ≤50 years or ≥50 polyps (of any histology) at age ≤30 years	19	7 (36.8%)	34 (14-50)	24	3	1.2
Total	98	73 (74.4%)	23.5 (0.7-67)	166	43	1.5

a Skin cancer, cervical cancer, and lung cancer in smokers were not considered due to their predominantly nongenetic etiologies.

Ninety-eight patients were recruited from ERN GENTURIS reference centers: Radboud University Medical Center (the Netherlands, n = 37), University Hospital Bonn (Germany, n = 30), Catalan Institute of Oncology–IDIBELL (Spain, n = 19), and Ipatimup/ULSSJ (Portugal, n = 12), with additional contributions from centers in Slovenia, Latvia, Italy, and Portugal. Clinical features, inclusion criteria, genes analyzed in prior testing, and referring centers are detailed in the Data Supplement (Table S1). Most participants (n = 90) had previous noninformative multigene, exome, or single-gene analyses, whereas eight had not undergone testing because of local eligibility restrictions.

The ethics committee/institutional review board of the University of Tübingen gave ethical approval for the work under the SOLVE-RD project (ClinicalTrials.gov identifier: NCT03491280). All patients provided informed consent for the use and sharing of their anonymized data and material for research on the identification of the genetic cause of their clinical condition.

### Summary of Bioinformatic and Experimental Methods

WGS variant calling and annotation were performed using established RD-Connect pipelines,^12,13^ followed by variant prioritization based on allele frequency, predicted functional impact, and gene-phenotype relevance. Structural variants (SVs), copy number variants (CNVs), and noncoding variants were further analyzed using GreenDB,^14^ SpliceAI,^15^ and UTRannotator.^16^

Microsatellite instability (MSI) testing, immunohistochemistry (IHC) for mismatch repair (MMR) proteins, and RT-qPCR assays were conducted using standard protocols.

Experimental and bioinformatic procedures are detailed in the Data Supplement.

## RESULTS

### Clinical Characteristics of the Patients

Among the 98 patients, 9% (9 of 98) met the multitumor criterion (I), 71% (70 of 98) the early-onset cancer criterion (II), and 19% (19 of 98) the polyposis criterion (III; Table 1). The median age at first cancer or polyposis diagnosis was 23.5 years. Females comprised 74% (73 of 98) of the cohort. In total, 166 cancer diagnoses were reported, representing 43 unique tumor phenotypes. Breast cancer was the most frequent, accounting for 23.5% (39 of 166) of all diagnoses and found in 44% (4 of 9) of criterion I and 46% (32 of 70) of criterion II patients. Criterion I showed the highest phenotypic diversity, with 28 of the 43 tumor types and an average of 4.8 phenotypes per patient (median: 4; range: 3-8). Criterion III had the lowest phenotypic diversity, with only three phenotypes observed: polyposis (19 of 19 patients), colorectal cancer (2 of 19), and uveal melanoma (1 of 19).

### Pathogenic Variants in Phenotype-Associated Hereditary Cancer Genes

**FIG 1.**
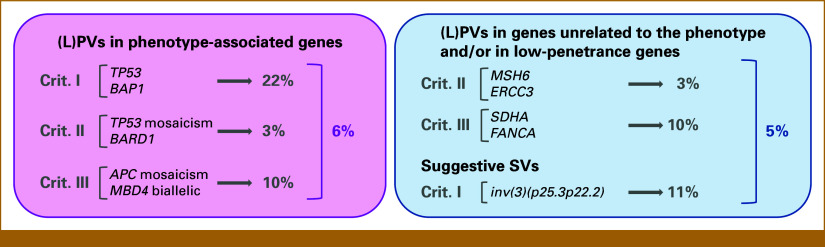
Key findings of the study. Left panel: Pathogenic and likely pathogenic variants in genes related to the patient's phenotype, categorized by fulfillment of clinical criteria. A definitive genetic diagnosis was made in 6% of patients, including 22% of those meeting criterion I, 3% of criterion II patients, and 10% of criterion III patients. Right panel: gPVs in known hereditary cancer genes a priori not related to the patient's phenotype and gPVs in low-penetrance genes. These variants were detected in 5% of patients. Crit, criterion; gPV, germline pathogenic variants; (L)PVs, pathogenic or likely pathogenic variants.

Pathogenic or likely pathogenic variants in hereditary cancer genes (low-risk genes excluded) matching the clinical phenotype of the corresponding patient were identified in six individuals (6% of the cohort). Two met criterion I, two criterion II, and two criterion III (Table 2; Fig 1). Variant classifications are detailed in the Data Supplement.

**TABLE 2.** Pathogenic and Likely Pathogenic Variants Identified in Known High-Penetrance Hereditary Cancer Genes That Likely Explain the Corresponding Patient's Clinical Phenotype

Gene [MANE transcript] HVGS Variant (coding and protein)	ACMG/AMP Variant Classification^a^	ClinVar Classification (No. of stars)	gnomAD v.4.1.0 MAF	Origin^b^ (VAF; sequencing reads)	Patient's ID and Phenotype (age at diagnosis, years)
Clinical criterion I					
*TP53* [NM_000546.6] c.1010G>A; p.(Arg337His)	P	P/LP (2)	0.0003%	Germline (VAF:0.55; 22 of 40 reads)	P0500005: Lung adenocarcinoma (57), leiomyosarcoma (55), breast cancer (67), multiple myeloma (68)
*BAP1* [NM_004656.4] c.437+2T>C; p.?^c^	LP	Not reported	Not reported	Germline (VAF: 0.54; 15 of 28 reads)	P0509904: Vulvar cancer (36), hepatocellular cancer (45), anal cancer (55), renal cancer (73), bladder cancer (69), eye tumor
Clinical criterion II					
*TP53* [NM_000546.6] c.916C>T; p.(Arg306*)	P	P (2)	0.00006%	Mosaicism (VAF:0.27; 12 of 44 reads)	P0823495: Breast cancer (23)
*FANCM* [NM_020937.4] c.1491dup; p.(Gln498Thrfs*7)	LP	P/LP (2)	0.0027%	Germline? (VAF: 0.32; 14 of 44 reads)	
*BARD1* [NM_000465.4] c.1538T>G; p.(Leu513*)	LP	Conflicting (1) [2 P, 1 VUS]	Not reported	Germline (VAF: 0.44; 25 of 56 reads)	P0850668: 2 breast cancers (22, 38)
Clinical criterion III					
*APC* [NM_000038.6] c.1879_1882del; p.(Asn627Leufs*2)	P	P (2)	Not reported	Mosaicism (VAF:0.11, 5 of 48 reads)	P0983025: Adenomatous polyposis (47; >50 polyps)
Homozygous *MBD4* [NM_001276270.2] c.939dup; p.(Glu314Argfs*13)	LP	Conflicting (1) [3 P/LP, 1 VUS]	0.054%	Germline (VAF:1.00, 25 of 25 reads)	P0319639: Adenomatous colorectal polyposis (50; >50 polyps), colorectal cancer (52), uveal melanoma (61)

NOTE. The table includes the description, classification, population allele frequency, and suggested origin of the variant, as well as the patients' ID and clinical phenotype.

Abbreviations: ccRCC, clear cell renal cell carcinoma; LP, likely pathogenic; MAF, minor allele frequency; P, pathogenic; VAF, variant allele fraction; VUS, variant of uncertain significance; WGS, whole-genome sequencing.

^a^ ACMG/AMP variant classification guidelines or ClinGen gene-specific recommendations if available. Rule codes and evidence used for the classification of the variants are given in the Data Supplement.

b Proposed origin based on VAF obtained from WGS of the patient's blood DNA. VAFs <30% were considered potential mosaicisms.

^c^ SpliceAI masked Δ score: 0.97.

*TP53* c.1010G>A p.(Arg337His) was found in a Portuguese female (P0500005) diagnosed with four cancers: lung adenocarcinoma (57 years), leiomyosarcoma (55 years), breast cancer (67 years), and multiple myeloma (68 years). This pathogenic variant, of incomplete penetrance and prevalent in Brazil, is identified in carriers of a founder haplotype of Lusitanian/Hispanic origin.^17^ In a cohort of 242 carriers, approximately 75% had breast cancer, soft tissue sarcoma, or lung cancer—aligning with this patient's phenotype.^18^

*TP53* c.916C>T p.(Arg306*) was identified at a variant allele fraction (VAF) of 27% in a 23-year-old female with breast cancer (P0823495), indicating likely somatic mosaicism (Clonal hematopoiesis of undetermined potential was unlikely due to age and treatment history). She also carried *FANCM* c.1491dup p.(Gln498Thrfs*7), a likely pathogenic variant associated with moderate breast cancer risk.^19-21^

*BAP1* c.437+2T>C, a splice site variant (donor loss: SpliceAI-masked Δ score = 0.97), was found in a female (P0509904) with multiple tumors: eye tumor, hepatocellular carcinoma (45 years), multifocal bladder carcinoma (69 years), and chromophobe renal carcinoma (73 years); all these are part of the phenotypic spectrum of the *BAP1* tumor predisposition syndrome.^22^ She also had vulvar and anal carcinomas (36 and 55 years), not typically linked to *BAP1*. Her son had cancer of unknown primary at age 49 years.

*BARD1* c.1538T>G p.(Leu513*) was identified in a female patient diagnosed with two primary breast cancers at age 22 and 38 years (P0850668). *BARD1* had not been included in the panel used for genetic testing at the center of origin. BARD1 interacts with BRCA1 in the homologous recombination DNA repair pathway, and the gene is considered a moderate-penetrance gene for breast cancer.^20,23^

*APC* c.1879_1882del p.(Asn627Leufs*2), with a VAF of 10% (5 of 48 reads) in WGS, was identified in a 47-year-old patient with adenomatous polyposis (P0983025), suggesting postzygotic somatic mosaicism. On rereview of the multigene panel sequencing data from the center of origin, a VAF of 2.8% (19 of 659 reads) was observed. It had been missed because of stringent VAF filters; presence was later confirmed using SNuPE.^24^

Homozygous *MBD4* c.939dup p.(Glu314Argfs*13) was found in a patient (P0319639) with polyposis (50 years), colorectal cancer (52 years), and uveal melanoma (61 years). This variant has been previously reported in patients with the recessive *MBD4*-associated neoplasia syndrome, characterized by increased risk of AML, GI polyposis, and colorectal cancer and, to a lesser extent, uveal melanoma and schwannomas.^25,26^ This gene had not been included in earlier panel testing.

### Potentially Relevant SNVs and Indels and Pathogenic Variants in Nonphenotype-Associated or Low-Penetrance Genes

All prioritized single nucleotide variants (SNVs) and small insertions and deletions (indels) in hereditary cancer genes across the 98 patients are listed in the Data Supplement (Table S1); this section focuses on (likely) pathogenic variants and phenotype-relevant VUS (Table 3).

**TABLE 3.** SNVs and Indels Classified as Pathogenic or Likely Pathogenic Variants That a Priori Do Not Explain the Corresponding Patient's Phenotype or Occur in Low-Penetrance Genes, and VUS of Interest

Gene [MANE transcript] HVGS Variant (coding and protein)	ACMG/AMP Variant Classification^a^	ClinVar Classification (No. of stars)	gnomAD v.4.1.0 MAF	Origin^b^ (VAF; sequencing reads)	Patient's ID and Phenotype (age at diagnosis, years)
Clinical criterion II					
*ERCC3* [NM_000122.2] c.325C>T; p.(Arg109*)	LP	P/LP (2)	0.0343%	Germline (VAF: 0.51; 19 of 37 reads)	P0088062: Breast cancer (invasive ductal carcinoma) (20)
*MSH6* [NM_000179.3]: c.3722G>A; p.(Cys1241Tyr)	LP	Conflicting (1) [2 P, 1 VUS]	Not reported	Germline (VAF: 0.41; 14 of 34 reads)	P0488786: Papillary thyroid cancer (19)
*ATM* [NM_000051.4]: c.8187A>C; p.(Gln2729His)	VUS	VUS (2)	0.0008%	Germline (VAF: 0.50; 16 of 32 reads)	P0283863: Breast cancer (invasive ductal carcinoma) (23)
*BRIP1* [NM_032043.3]: c.2076G>C; p.(Leu692Phe)	VUS	Not reported	Not reported	Germline? (VAF: 0.36, 12 of 33 reads)	P0291290: Breast cancer (22)
*FANCM* [NM_020937.4]*:* c.5791C>T; p.(Gly1906Alafs12*)	VUS	P/LP (2)	0.0951% (4 homozygotes)	Germline (VAF: 0.50; 23 of 46 reads)	P0019693: Ovarian cancer (21), breast cancer (56), colorectal cancer (61)
*POLD1* [NM_002691.4]: c.910T>C; p.(Trp304Arg)	VUS	VUS (1)	0.00006%	Germline (VAF: 0.50; 12 of 24 reads)	P0242687: Breast cancer (24), ovarian cancer (57), colorectal cancer (68)
Clinical criterion III					
*SDHA* [NM_004168.4]: c.688del; p.(Glu230Serfs*10)	LP	P/LP (2)	0.0009	Germline (VAF: 0.58; 18 of 31 reads)	P0179987: Adenomatous polyposis (26): 100-500 polyps
*FANCA* [NM_000135.4]: c.862G>T; p.(Glu288*)	LP	P (2)	0.0075	Germline (VAF: 0.54; 20 of 37 reads)	P0429955: Adenomatous polyposis (14): 100-500 polyps

NOTE. The table includes the description, classification, population allele frequency, and suggested origin of the variant, as well as the patients' clinical phenotypes.

Abbreviations: Indels, small insertions and deletions; LP, likely pathogenic; MAF, minor allele frequency; P, pathogenic; SNV, single nucleotide variant; VAF, variant allele fraction; VUS, variant of uncertain significance; WGS, whole-genome sequencing.

^a^ ClinGen gene-specific recommendations or ACMG/AMP variant classification guidelines if not available. Rule codes and evidence used for the classification of the variants are given in the Data Supplement.

^b^ Proposed origin based on VAF obtained from WGS of the patient's blood DNA. VAFs < 30% were considered potential mosaicisms.

A truncating *ERCC3* variant, c.325C>T p.(Arg109*), recurrent in Ashkenazi Jewish populations, was identified in a 20-year-old patient with breast cancer (P0088062). Heterozygous *ERCC3* gPVs and, in particular, this variant, have been associated with a low/moderate breast cancer risk.^27,28^ The patient was also heterozygous for a *FANCM* VUS, c.1667A>G; p.(Asp556Gly).

*MSH6* c.3722G>A p.(Cys1241Tyr), likely pathogenic, was identified in a 19-year-old male patient with papillary thyroid cancer (P0488786), validated by Sanger sequencing. Although thyroid cancer is not typically associated with Lynch syndrome, several cases of papillary thyroid cancer in patients with Lynch syndrome have been reported.^29-32^ *MSH6* IHC on normal and tumor thyroid tissues was inconclusive, hindering the establishment of a causal link. Notably, the patient had a second-degree relative with endometrial cancer showing MSH6 protein loss. The proband also carried a VUS in *SDHA* (c.1799G>A; p.Arg600Gln), a gene with no established link with thyroid cancer.

*ATM* c.8187A>C p.(Gln2729His) and *BRIP1* c.2076G>C p.(Leu692Phe), both VUSs, were identified in two patients with breast cancer diagnosed in their early 20s (P0283863 and P0291290, respectively). *ATM* gPVs are associated with increased risk of several cancers, particularly invasive ductal breast cancer,^33^ which is the tumor type diagnosed in P0283863. *BRIP1* gPVs mainly confer (moderate) ovarian cancer risk, with limited evidence for breast cancer.^34,35^

*FANCM* c.5791C>T p.(Gly1906Alafs12*) (MAF: 0.1%; gnomAD v.4.1.0) was found in a patient with ovarian (21 years), breast (56 years), and colorectal (61 years) cancers (P0019693). This nonsense variant causes exon skipping, predicted to encode a protein that incorporates 11 additional residues, leading to a premature termination of translation that results in the loss of 132 amino acids from the protein C-terminus. It affects DNA repair activity and has been associated with moderately increased breast cancer risk.^36^ The patient's multitumor phenotype suggests potential additional genetic or nongenetic risk factors.

*POLD1* c.910T>C p.(Trp304Arg), a VUS in the exonuclease domain of polymerase δ, was found in a patient with breast (24 years), ovarian (57 years), and colorectal (68 years) cancers (P0242687). These cancers align with the *POLE/POLD1*-associated cancer syndrome.^37^ Tumor mutational analysis would clarify the variant's pathogenicity,^37,38^ but no tumor material was available.

Two likely pathogenic heterozygous variants were found in patients with phenotypes not typically linked to the associated gene: *SDHA* c.688del p.(Glu230Serfs10) in a 46-year-old patient with adenomatous polyposis (P0179987) and *FANCA* c.862G>T p.(Glu288) in a 14-year-old patient with polyposis (P0429955). Neither patient exhibited clinical features associated with these genes. Because of SDHA's low penetrance and the uncertain role of heterozygous *FANCA* variants in cancer predisposition, these variants were not reported to the patients.

### Structural Variants of Potential Interest

**FIG 2.**
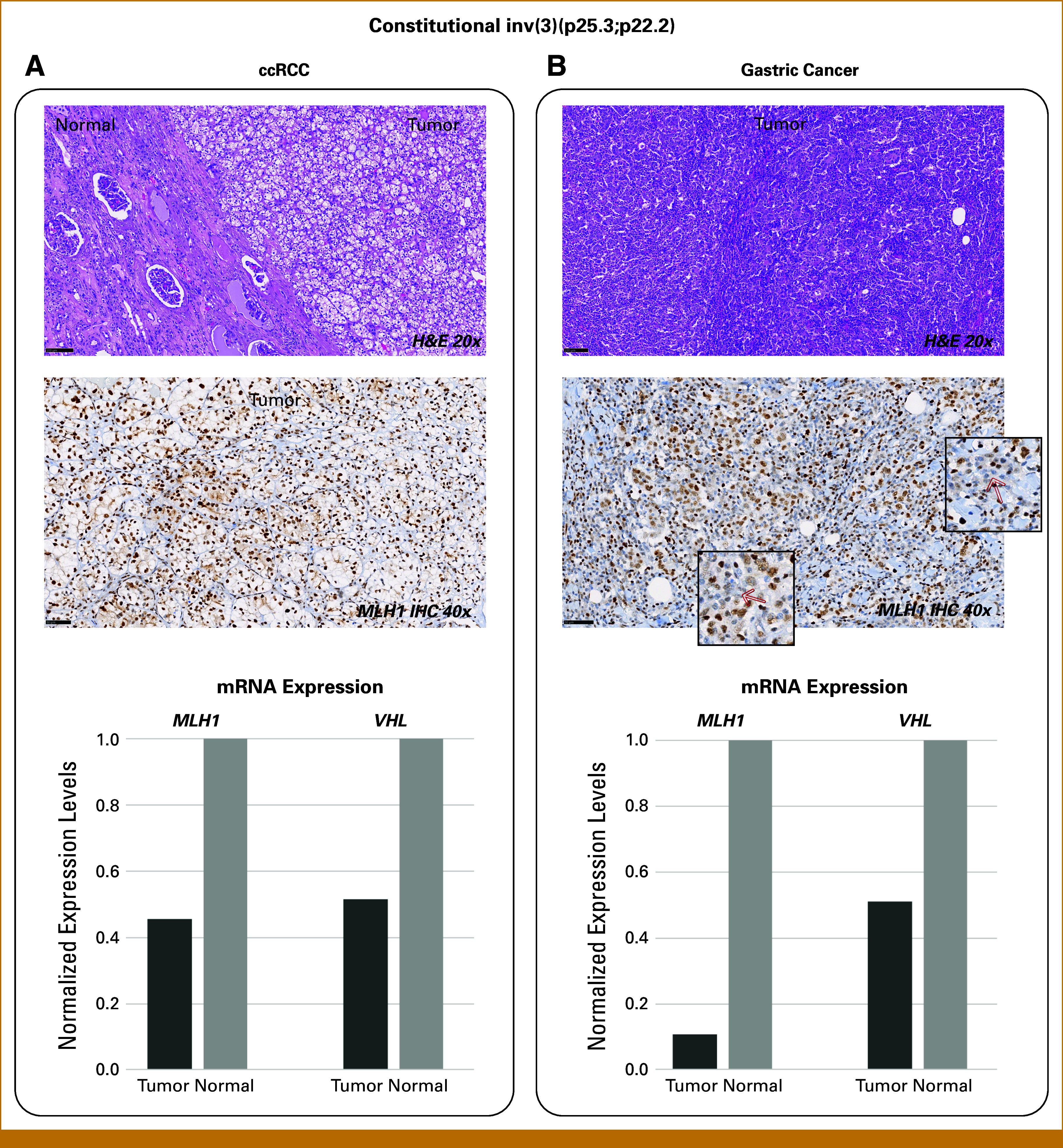
Characteristics of the (A) ccRCC and (B) gastric cancer of the patient with constitutional inv(3)(p25.3;p22.2) [P0085308]. From top to bottom: H&E staining, IHC of MLH1 protein in tumor tissue, and *MLH1* and *VHL* mRNA expression in tumor and adjacent normal tissue. MLH1 IHC staining in the gastric cancer showed focal loss of MLH1 expression (magnified regions, red arrows). ccRCC, clear cell renal cell carcinoma; H&E, hematoxylin and eosin; IHC, immunohistochemistry.

A 29-Mb inversion on chromosome 3 (inv(3)(p25.3;p22.2)) was found in patient P0085308, who was diagnosed with clear cell renal cell carcinoma (ccRCC; stage pT1a), two lung cancers (squamous and small cell; nonsmoker), and gastric carcinoma with lymphoid stroma, a subtype associated with somatic DNA MMR deficiency (Data Supplement, Table S3). The inversion disrupts *ETD5* and *SCN11A*, genes not linked to cancer, but it also affects *VHL*, a key ccRCC predisposition gene, and *MLH1*, associated with Lynch syndrome, which may elevate gastric cancer risk. Similar constitutional chromosomal rearrangements involving the short arm of chromosome 3 have been observed in families/patients with ccRCC.^39-56^ The presence of gastric cancer has been observed when the rearrangement also includes *MLH1*.^57-59^ Tumor analysis using formalin-fixed paraffin-embedded gastric and renal samples (Fig 2) revealed no MSI or loss of MLH1 and/or PMS2 protein expression by IHC (Figs 2A and 2B; Data Supplement, Fig S1). However, the gastric tumor showed heterogeneous MLH1 protein expression, with reduced nuclear expression in some cells and focal areas of expression loss (Fig 2B). Both tumors exhibited significant downregulation of *MLH1* and *VHL* mRNA expression compared with adjacent normal tissue (Figs 2A and 2B).

Eight additional rare chromosomal inversions (MAF ≤0.1% in the SOLVE-RD cohort of 2,992 individuals) involving any of the 127 hereditary cancer genes analyzed were identified in eight patients (Data Supplement, Table S3). Three had breakpoints in intergenic regions, and five disrupted genes not previously linked to cancer. Notably, two young patients with breast cancer (P0831645 and P0823495—the latter heterozygous for *TP53* and *FANCM* gPVs) had an inversion affecting the same genomic region on chromosome 2, inv(2)(p11.2;q31.1). This region includes *ERCC3*, associated with moderate breast cancer risk. The role of this inversion in early-onset breast cancer remains unclear. Aside from inv(3)(p25.3;p22.2) and inv(2)(p11.2;q31.1), no other structural variants overlapped phenotype-related genes. Further validation is needed to confirm the presence of these inversions.

### Noncoding Variants in Phenotype-Specific Hereditary Cancer Genes With a Potential Functional Effect

Following the prioritization criteria defined in the Data Supplement, no noncoding variants with SpliceAI Δ ≥ 0.2—excluding canonical splicing effects—were identified. A total of 22 noncoding variants with a GreenDB constraint of ≥0.90 were detected in 15 patients (Data Supplement, Table S4).

Several variants were considered of particular interest because of their gene-phenotype concordance: a *BRIP1* promoter variant (c.2097+15511T>G) in a 20-year-old patient with breast cancer (P0088062), a *RAD51C* enhancer variant (c.904+482C>G) in a 25-year-old patient with ovarian cancer (P0267036), and a potentially regulatory *NF1* enhancer variant (c.888+2091G>A) in a patient (P0082961) with multiple tumors—including parathyroid adenoma, leiomyosarcoma, cutaneous melanoma, basal cell carcinoma, and prostate cancer—who also carried two additional potentially regulatory *NF1* variants (GreenDB constraint > 0.70). Although these tumor types are not considered bona fide *NF1*-associated tumors, some occur more frequently in *NF1* individuals than in the general population.^60^ A *CDH1* regulatory variant (c.163+16686T>A; enhancer/promoter) was identified in a patient with breast cancer and epithelioid hemangioendothelioma (P0810159), although typically the variants associated with increased cancer risk (mostly gastric and breast cancers) are truncating gPVs.^61^ Finally, a *BMPR1A* enhancer variant (c.-267-25558G>A) was found in a 35-year-old patient with adenomatous polyposis. Although *BMPR1A* is classically associated with juvenile polyposis syndrome, recent studies suggest its involvement in adenomatous polyps.^62,63^

## DISCUSSION

Genetic testing using multigene panels followed by phenotype-directed analysis is cost-effective but may fail to identify the underlying genetic cause in some patients with strong suspicion of hereditary cancer. To address this, ERN GENTURIS, through the Horizon 2020-supported SOLVE-RD project,^10^ selected cases with highly suspicious phenotypes but no prior genetic diagnosis. Inclusion criteria comprised multiple primary tumors (≥4 or ≥3 if ≥ 2 were rare), very early onset (≤25 years) of adult-type cancers, or early-onset polyposis. To capture coding and noncoding regions of the genome, including all known hereditary cancer genes, and facilitate the identification of CNVs and SVs, short-read WGS was performed on 98 unrelated patients, primarily meeting the early-onset criterion (approximately 70%). Analysis focused on a curated panel of 127 hereditary cancer genes, including all ClinGen-validated genes. Definitive, phenotype-concordant diagnoses were established in six patients, increasing the diagnostic yield by 6%. These included gPVs in high- or moderate-/high-penetrance genes. Solved cases were most frequent among multitumor phenotypes (22%), followed by polyposis (10%) and early-onset cancers (3%; Fig 1). In addition, an *MSH6* gPV was identified in a patient with papillary thyroid cancer.

Several factors may explain why standard testing missed these diagnoses: low-level mosaicism filtered out because of stringent VAF thresholds, involvement of newly recognized or rarely tested genes, reclassification of variants as pathogenic, undetectable SVs/CNVs in panel testing, and coverage gaps or incomplete clinical data guiding gene selection.

Somatic postzygotic mosaicism was detected in two patients. The first, a 23-year-old patient with breast cancer initially tested only for *BRCA1/2*, carried a potential mosaic *TP53* pathogenic variant (VAF 27%). The second, a patient with polyposis, had *APC* mosaicism (VAF 11%), which was missed by multigene panel sequencing because of a low VAF (2.8%). Compared with WGS, gene panel sequencing—particularly with relaxed or no VAF filters—is more effective for detecting mosaicism because of its higher coverage depth. *APC* mosaicism is relatively common in adenomatous polyposis,^64^ supporting the use of lower VAF thresholds in germline analysis and the testing of multiple adenomas/tumors, as the variant may be absent in blood but present in tissue-specific cells, particularly in tumor cells.^24,65,73^ Although no deep intronic *APC* variants affecting splicing were identified in this study, analysis of *APC*'s noncoding regions should be considered in unsolved adenomatous polyposis cases.^66-69^ Mosaicisms and deep intronic splicing-altering variants may also occur in other hereditary cancer genes.

A patient with adenomatous polyposis (P0850712; >70 adenomas by age 25) underwent tumor sequencing on three polyps and normal colon mucosa during the course of this study. A pathogenic *APC* variant, c.3394G>T; p.(Glu1132*), was identified in all three polyps (VAFs: 34%-35%) and normal colon mucosa (VAF: 4%), but not in blood, suggesting postzygotic somatic mosaicism. This highlights the importance of testing target tissues (multiple polyps, normal mucosa) in patients with unexplained adenomatous polyposis as *APC* mosaicism is a common cause.^64,73,74^

In addition to the identification of gPVs, VUSs were detected in genes of interest: *POLD1* (breast, ovarian, colorectal cancers) and *ATM* and *BRIP1* (early-onset breast cancer). An SV consisting of a 29-Mb inversion on chromosome 3, inv(3)(p25.3;p22.2), was identified in a patient with ccRCC, gastric cancer, and two lung cancers. The inversion spans *VHL* (ccRCC predisposition) and *MLH1* (Lynch syndrome gene), suggesting a possible causal link. Tumor analysis showed no evidence of MSI or loss of MLH1 protein expression, except for focal loss of nuclear expression in the gastric tumor. However, a significant reduction in *MLH1* and *VHL* mRNA expression was observed in both the ccRCC and gastric cancer. Taking the available evidence into consideration, the inversion is currently classified as of uncertain significance. Multiple chromosomal rearrangements involving the short arm of chromosome 3, always including *VHL*, have been associated with high risk of adult-onset ccRCC and, in some instances, also of gastric cancer.^45,56^ The majority of the ccRCCs developed in the context of this cancer syndrome show somatic loss of the derivative chromosome harboring the 3p fragment and often a somatic mutation in the *VHL* gene in the remaining allele, demonstrating complete abrogation of *VHL*.

Heterozygous gPVs were identified in *FANCA*, *ERCC3*, and *FANCM*. These genes are associated with recessive disorders, and in heterozygosity, the cancer risk they pose—such as for breast cancer—is at most moderate or low,^27,28,70,71^ which is considered insufficient to affect clinical management or alter surveillance strategies. In addition, a gPV in *SDHA* was identified in a patient with polyposis. This gene is associated with paraganglioma predisposition; however, its low penetrance also limits its clinical utility.^72^

Excluding low-/moderate-risk genes or those with dubious associations (eg, *ERCC3*, *FANCM*, *FANCA*, *SDHA*), six pathogenic or likely pathogenic variants were identified. Five of these affected genes had not been considered in the initial germline testing, either because of their exclusion from the panel or phenotype-based selection. These included *TP53* (c.1010G>A; p.(Arg337His), c.916C>T; p.(Arg306*)), homozygous *MBD4* (c.939dupA; p.(Glu314Argfs13)), *MSH6* (c.3722G>A; p.(Cys1241Tyr)), *BAP1* (c.437+2T>C), and *BARD1* (c.1538T>G; p.(Leu513)), as well as a missed mosaicism in *APC* (c.1879_1882delAACA). These variants would have been identified by whole-exome or multigene panel sequencing if the analysis had included all relevant hereditary cancer genes and applied a less stringent VAF threshold to detect mosaic cases. While WGS did not yield additional clinically relevant findings, it identified a chromosome 3 inversion involving *MLH1* and *VHL* in a patient with ccRCC and gastric and lung cancers, which is currently classified as a variant of uncertain significance.

This study also sought to identify noncoding variants that might affect splicing or gene expression in phenotype-related genes. Although no variants were predicted to alter splicing, 22 potentially regulatory variants were detected in 15 patients, some in genes with known relevance to their phenotypes. The functional impact of these variants remains uncertain and should be validated through allele-specific expression analyses, reporter assays, or in vitro studies, ideally considering tissue-specific regulatory effects. Tumor sequencing could further support their relevance by revealing somatic second hits or mutational signatures indicative of DNA repair defects.

All identified gPVs in this cohort were located within coding regions of known hereditary cancer genes, suggesting that resequencing or reanalysis with extended gene panels or whole-exome sequencing would have resolved all cases. Nonetheless, inclusion of noncoding regions remains relevant as these may harbor gPVs affecting splicing or regulatory elements. WGS proved to be particularly valuable by uncovering a suggestive chromosome 3 inversion and regulatory variants. Unresolved cases may also involve somatic mosaicism with low VAF in hematopoietic cells, requiring less stringent or tissue-specific analyses. We recommend retrospective reanalysis or resequencing of unresolved cases, integrating the analysis of both coding and noncoding regions and considering postzygotic mosaicism. It should be noted that, despite its advantages, WGS—because of its lower coverage depth compared with targeted multigene panels—may fail to detect some clinically relevant variants.

In conclusion, resequencing of undiagnosed patients with strongly suggestive phenotypes—performed in our study using WGS—increased the diagnostic yield by 6%, with an additional 5% showing variants of high interest (Fig 1). Our approach enabled the identification of coding gPVs, as well as candidate coding and noncoding variants and SVs in phenotype-associated genes. To validate the relevance of coding and noncoding VUS, further tumor mutational analyses, cosegregation studies, and functional assays will be essential. We recommend retrospective reanalysis or resequencing of unresolved cases, incorporating both coding and noncoding regions, with particular attention to SVs, postzygotic mosaicism, and deep intronic splicing-altering variants, to enhance diagnostic accuracy and gain deeper insights into the underlying genetic mechanisms.

While this study focuses on known cancer predisposition genes and diagnostic aspects, future research will explore, through broader analysis of the generated WGS data, their potential for the identification of new hereditary cancer genes.

## Data Availability

A data sharing statement provided by the authors is available with this article at DOI https://doi.org/10.1200/PO-25-00354. All raw and processed data files (FASTQs, CRAMs, CRAIs, VCFs) are available at the European Genome-Phenome Archive (https://ega-archive.org/, data set EGAD00001015604 under the Solve-RD study EGAS00001003851), following approval from the Solve-RD Data Access Committee.
